# Ocular Manifestations of von Hippel-Lindau Disease

**DOI:** 10.7759/cureus.5319

**Published:** 2019-08-04

**Authors:** Misty D Ruppert, Meredith Gavin, Kelly T Mitchell, Alan N Peiris

**Affiliations:** 1 Internal Medicine, Texas Tech University Health Sciences Center, Lubbock, USA; 2 Ophthalmology, Texas Tech University Health Sciences Center, Lubbock, USA

**Keywords:** von hippel-lindau, hemangioblastoma, retinal capillary hemangioma, ocular angiomatosis, vhl gene

## Abstract

In this review article, we aimed to analyze the available data on the ocular manifestations of von Hippel-Lindau (VHL) disease. In this disease, the VHL protein becomes inactivated by germline mutations of the *VHL* tumor suppressor gene on chromosome 3p25-26, resulting in an overproduction of VEGF in non-hypoxic conditions. Ocular manifestations are expected in roughly half of VHL patients. Retinal capillary hemangioblastomas (RCHs) are the most commonly observed tumors in VHL and are often the initial manifestation of the disease. Ablative therapy, surgical resection, and pharmacotherapy have been implemented to control tumors. Left untreated, RCHs will often enlarge, emphasizing the importance of early diagnosis and treatment to preserve vision. Complications of enlarging peripheral or optic nerve tumors may be severe. Large RCHs may disrupt normal retinal architecture, eventually leading to exudative retinal detachment. Rarely, non-retinal manifestations, such as neovascularization of the iris or cornea, may progress to neovascular glaucoma and vision loss. Ablative therapy of larger tumors carries increasing risks and offers limited success, often necessitating surgical resection. Because this life-threatening disease is not routinely encountered in clinical practice, clinicians will benefit from our review which brings awareness to the ocular presentation of VHL and lifelong screening recommendations for diagnosed patients.

## Introduction and background

Von Hippel-Lindau (VHL) disease, also known as familial cerebello-retinal angiomatosis, is one of the neurocutaneous syndromes or phakomatoses. VHL affects approximately one in 36,000 live births and is an autosomal dominant condition characterized by the development of tumors, most commonly hemangioblastomas of the retina and central nervous system (CNS), clear cell renal carcinoma, and pheochromocytoma [1].

This disease has been categorized into types 1 and 2. Type 1 is characterized by retinal angiomas, CNS hemangioblastomas, renal cell carcinomas, pancreatic cysts, and neuroendocrine tumors, whereas type 2 is characterized by pheochromocytomas, retinal angiomas, and CNS hemangioblastomas [2]. Ocular manifestations are expected in roughly half of the patients affected by VHL [3].

In this review, we describe the ocular manifestations of VHL, which can be viewed as a progressive neurodegenerative disease [4].

## Review

Molecular and genetic basis

In VHL disease, the VHL protein becomes inactivated by germline mutations of *VHL* tumor suppressor gene on chromosome 3p25-26 [5]. The VHL protein is responsible for the ubiquitination and degradation of hypoxia-inducible factor-1 alpha (HIF-1 alpha), a transcription factor that induces vascular endothelial growth factor (VEGF) expression [6]. Inactivation of the *VHL* gene and subsequent overproduction of VEGF in normoxic conditions may be responsible for the increased angiogenesis seen in VHL [7].

The VHL protein may become inactivated by missense, frameshift, nonsense, in-frame deletions/insertions, large/complete deletions, and splice mutations [8]. In 1999, the lack of association between the position or type of mutation and the severity of ocular angiomatosis was reported [9]. However, in 2010, it was indicated that the location of missense mutations correlates significantly with the prevalence and phenotype of VHL ocular disease and is, therefore, a factor influencing the risk of vision impairment [10]. VHL patients with complete deletions reportedly develop ocular lesions less frequently than those with partial deletions, simple missense or nonsense mutations [3].

Regulation of the VHL protein is necessary for normal ocular growth and vascular development in the anterior chamber as well as maintenance of the retinal vasculature [11]. Ocular lesions in VHL have displayed up-regulated VEGF, and ocular fluid obtained from the anterior chamber in VHL patients contained significantly higher VEGF levels than in unaffected patients [3,12]. Additionally, increased endothelial cells found in tumors indicates that the VHL tumor suppressor gene plays a role in angiogenesis in VHL [12].

Although not routinely used for clinical diagnosis of VHL, VEGF and miRNA 210 may serve as biomarkers for disease activity [13]. Clusterin was suggested as a potential biomarker of ocular VHL disease after clusterin immunoreactivity was found to be decreased in retinal and the optic nerve hemangioblastomas [14]. Autophagy, inflammation, and upregulation of hypoxia-inducible factor 2 alpha are potential factors for aggressive tumors with resistance to multiple anti-VEGF and radiation treatments [15]. The avidity of VHL hemangioblastomas to somatostatin analogs suggests that these tumors demonstrate somatostatin receptor expression and offers a potential therapeutic target for tumors that have failed other treatments [16]. Somatostatin receptor imaging may become a useful future modality for the workup and management of certain VHL patients [17]. Expression of CXCR4 in retinal capillary hemangioblastomas (RCH) has been reported and may be targeted as an additional therapeutic attempt [18].

Ocular manifestations

Retinal Presentation and Visualization

Retinal capillary hemangioblastomas are well-recognized as the most commonly observed tumors in VHL and are commonly the initial manifestation of the disease [18,19]. The likelihood of retinal angioma development increases with age, reaching a probability of 80% in VHL patients over 80 years [5]. A recent study in Denmark found VHL to be the underlying cause in 84% of RCH cases, emphasizing the importance of VHL screening in patients diagnosed with RCH [20]. Hemangioblastomas occur in approximately 68% of VHL patients [9]. A large cross-sectional study reported that 42% of RCHs presented unilaterally and 58% presented bilaterally in VHL patients, with no association of age, gender, or laterality of involvement [21]. These tumors are often detected in the peripheral retina but may also occur in the juxtapapillary area [21]. A solitary juxtapapillary angioma finding should prompt a screen for VHL, which is best detected by molecular genetic diagnostics [22]. Retinal angiomas in VHL disease may be associated with true reactive diffuse massive retinal gliosis [23].

A patient with a VHL-associated RCH may initially present with a red or gray dot on the retina that is a few 100 microns in size [3]. In larger tumors, tortuous feeder vessels may become visible, and retinal edema and hard exudates may develop around the tumor and in the macula [3]. Unusual vascular hamartomas may also suggest VHL and can exist without retinal angiomas [24]. Fluorescein angiography may be helpful in visualizing these tumors, which appear as small vascular lesions often located in the superficial retina adjacent to the retinal vein [24]. Retinal hemangioblastomas associated with retinal non-perfusion have been described in VHL [25]. The use of specialized cameras and ultra-wide fluorescein angiography provide better detection of hemangioblastomas than conventional angiography and ophthalmoscopy [26]. Indocyanine angiography has also been mentioned for its possible investigative role in VHL ocular disease [27].

Non-retinal Manifestations

Other rare associations of VHL include neovascularization of the iris, known as rubeosis iridis, which develops secondary to longstanding exudative or tractional retinal detachment and can progress to neovascular glaucoma [3]. Enlarging peripheral or optic nerve lesions that disrupt normal retinal architecture put patients at risk for exudative retinal detachment and poor visual acuity [3]. Ultimately, corneal neovascularization and rubeosis iridis may lead to corneal perforation and vision loss [28].

Established treatment strategies

Ablative Therapy

Small peripheral RCHs less than 1.5 mm often remain stabile and can be initially observed [29]. If these tumors progress, ablative treatments in the form of argon laser photocoagulation and cryotherapy have been effective treatments [29-32]. Multiple laser photocoagulation sessions and, occasionally, additional cryotherapy after laser photocoagulation may be necessary to achieve RCH inactivation [32]. Other ablative therapies have included photodynamic therapy, transpupillary thermotherapy, plaque radiotherapy, external beam radiotherapy, and vitreoretinal surgical ablation [19]. As RCHs enlarge, it becomes increasingly difficult to destroy these lesions with photocoagulation or cryotherapy, and these treatments carry increased risks. Retinal hard exudates and macular edema are potential complications of photocoagulation and cryotherapy of peripheral RCHs [3]. Corticosteroids given preoperatively or postoperatively have been considered in an attempt to mitigate these adverse treatment effects [3].

Adjunctive endolaser photocoagulation at vitrectomy has been effective in severe cases, although the RCH recurrence rate is high [33]. Although not a routine therapy, verteporfin infusion in photodynamic therapy has recently been proven a suppressor of yes-associated protein and has been used for refractory retinal lesions [34-35]. Ruthenium-106 brachytherapy has been implemented for very large tumors, especially if there is no preoperative exudative retinal detachment [36]. External beam radiation may be considered in refractory cases. Patients treated with this therapy have reportedly experienced improved vision, decreased RCH volume and retinal detachment stabilization [37].

Observation is the initial management of juxtapapillary RCHs as these lesions can remain relatively static for long periods of time [29]. Radiotherapy or cryotherapy of juxtapapillary RCHs that lie close to the optic nerve may result in visual loss; in such cases, photodynamic therapy with verteporfin may be considered [38]. Laser photocoagulation of lesions next to the optic nerve may result in central scotomas [39]. Photodynamic therapy of these lesions has shown poor success [40].

Surgical Therapy

Because ablative therapy of large tumors carries increasingly significant risks, surgical resection may become necessary. Tractional retinal detachment may occur from the contraction of RCH fibrovascular lesions [3]. Pars plana vitrectomy along with a relaxing retinectomy may preserve or improve vision in the presence of tractional retinal detachment in advanced VHL ocular disease [41-42]. Vitrectomy with surgical resection has improved vision in patients with large RCHs, but the long-term recurrence rate after surgical resection is unfortunately very high [33,43-44].

Pharmacotherapy

Tumors located close to the macula and optic nerve are difficult to treat with ablative therapy. Intravitreous VEGF antagonists such as intravitreal ranibizumab may decrease RCH exudation, especially in small lesions with minimal exudation [45-46]. However, ranibizumab monotherapy has minimal efficacy for the majority of VHL-related RCHs [46]. Intravitreal bevacizumab improved visual acuity, resolved exudates, and edema, and stabilized structural lesions in a patient treated over 60 months [47]. Intravitreal propranolol has also proven beneficial in some patients with RCHs, and oral administration of propranolol has decreased exudation [13,48]. Treatment with oral sunitinib, a tyrosine kinase inhibitor that blocks both VEGF and platelet-derived growth factor receptors, improved retinal edema in some patients but had multiple adverse effects [49].

Screening

VHL patients require routine lifetime screening and frequent surveillance of any discovered lesions. The VHL Alliance has provided detailed screening guidelines, which are summarized in Table 1 [50].

**Table 1:** 2017 screening guidelines from the VHL Alliance VHL, von Hippel-Lindau; [50]

Age (years)	Screening Recommendations
1 to 4	Begin yearly eye examinations with the indirect ophthalmoscope to rule out retinal lesions. Begin annual neurological screening for nystagmus, strabismus, white pupil, vision and hearing impairment, and blood pressure abnormalities.
5 to 15	Continue the routine screening described in the previous age group. Begin lifelong biochemical screening for fractionated metanephrines, including normetanephrine and plasma free metanephrines at age 5. Begin audiology assessments every 2 to 3 years or yearly in the presence of hearing impairment, tinnitus, or vertigo.
16 and older	Continue the routine screening described in the previous age groups. Begin yearly quality contrast and noncontrast ultrasounds to rule out renal, pancreatic and adrenal tumors. Begin MRI scans (no less than a 1.5T MRI performed with and without contrast) every 2 to 3 years of the brain, cervical, thoracic, and lumbar spine with visualization of the posterior fossa and inner ear/petrous temporal bone to rule out endolymphatic sac tumors and neuraxis hemangioblastomas.

## Conclusions

The majority of patients with VHL maintain stable ocular function with treatment; however, the disease may progress and cause vision loss. Without treatment, tumors often enlarge over time, and large RCHs increase the risk of exudative and traction retinal detachment. VHL disease is a life-threatening, progressive disorder that must be followed closely, not only for prevention and treatment of ocular manifestations, but also for surveillance of systemic findings of the disease. It is imperative that clinicians identify VHL patients for early counseling, close follow-up, and proactive treatment. Retinal angiomas or hamartomas should immediately prompt further workup to rule out VHL. Regular screening to identify the development of new angiomas and early diagnosis of VHL may facilitate greater treatment success. Early treatment, along with newer medical therapies, offers hope in the context of a disease that can result in profound visual loss.

## References

[1] Maher ER, Neumann HP, Richard S (2011). Von Hippel-Lindau disease: a clinical and scientific review. Eur J Hum Genet.

[2] Varshney N, Kebede AA, Owusu-Dapaah H (2017). A review of Von Hippel-Lindau syndrome. J Kidney Cancer VHL.

[3] Chew EY (2005). Ocular manifestations of von Hippel-Lindau disease: clinical and genetic investigations. Trans Am Ophthalmol Soc.

[4] Wong WT, Yeh S, Chan CC (2008). Retinal vascular proliferation as an ocular manifestation of von Hippel-Lindau disease. Arch Ophthalmol.

[5] Wittebol-Post D, Hes FJ, Lips CJ (1998). The eye in von Hippel-Lindau disease. Long-term follow-up of screening and treatment: recommendations. J Intern Med.

[6] Na XI, Wu G, Ryan CK (2003). Overproduction of vascular endothelial growth factor related to von Hippel-Lindau tumor suppressor gene mutations and hypoxia-inducible factor—1α expression in renal cell carcinomas. J Urol.

[7] Iliopoulos O, Levy AP, Jiang C (1996). Negative regulation of hypoxia-inducible genes by the von Hippel-Lindau protein. Proc Natl Acad Sci U S A.

[8] Nordstrom-O'Brien M, van der Luijt RB, van Rooijen E (2010). Genetic analysis of von Hippel-Lindau disease. Hum Mutat.

[9] Webster AR, Maher ER, Moore AT (1999). Clinical characteristics of ocular angiomatosis in von Hippel-Lindau disease and correlation with germline mutation. Arch Ophthalmol.

[10] Mettu P, Agrón E, Samtani S (2010). Genotype-phenotype correlation in ocular von Hippel-Lindau (VHL) disease: the effect of missense mutation position on ocular VHL phenotype. Invest Ophthalmol Vis Sci.

[11] Lange CAK, Luhmann UFO, Mowat FM (2012). Von Hippel-Lindau protein in the RPE is essential for normal ocular growth and vascular development. Development.

[12] Los M, Aarsman CJ, Terpstra L (1997). Elevated ocular levels of vascular endothelial growth factor in patients with von Hippel-Lindau disease. Ann Oncol.

[13] Albiñana V, Escribano RMJ, Soler I (2017). Repurposing propranolol as a drug for the treatment of retinal haemangioblastomas in von Hippel-Lindau disease. Orphanet J Rare Dis.

[14] Zhou M, Shen D, Head JE (2007). Ocular clusterin expression in von Hippel-Lindau disease. Mol Vis.

[15] Wang Y, Abu-Asab MS, Shen D (2014). Upregulation of hypoxia-inducible factors and autophagy in von Hippel-Lindau-associated retinal hemangioblastoma. Graefes Arch Clin Exp Ophthalmol.

[16] Sizdahkhani S, Feldman MJ, Piazza MG (2017). Somatostatin receptor expression on von Hippel-Lindau-associated hemangioblastomas offers novel therapeutic target. Sci Rep.

[17] Papadakis GZ, Millo C, Sadowski SM (2016). Endolymphatic Sac Tumor Showing Increased Activity on 68Ga DOTATATE PET/CT. Clin Nucl Med.

[18] Liang X, Shen D, Huang Y (2007). Molecular pathology and CXCR4 expression in surgically excised retinal hemangioblastomas associated with von Hippel-Lindau disease. Ophthalmology.

[19] Şahin Atik S, Solmaz AE, Öztaş Z (2017). Von Hippel-Lindau disease: the importance of retinal hemangioblastomas in diagnosis. Turk J Ophthalmol.

[20] Binderup MLM, Stendell A-S, Galanakis M (2018). Retinal hemangioblastoma: prevalence, incidence and frequency of underlying von Hippel-Lindau disease. Br J Ophthalmol.

[21] Wong WT, Agrón E, Coleman HR (2008). Clinical characterization of retinal capillary hemangioblastomas in a large population of patients with von Hippel-Lindau disease. Ophthalmology.

[22] Kreusel KM, Bechrakis NE, Neumann HP (2007). Solitary juxtapapillary capillary retinal angioma and von Hippel-Lindau disease. Can J Ophthalmol.

[23] Vortmeyer AO, Chan CC, Chew EY (1999). Morphologic and genetic analysis of retinal angioma associated with massive gliosis in a patient with von Hippel-Lindau disease. Graefes Arch Clin Exp Ophthalmol.

[24] Schmidt D, Neumann HP (1995). Retinal vascular hamartoma in von Hippel-Lindau disease. Arch Ophthalmol.

[25] Pulido JS, Dalvin LA, Olsen TW (2018). Peripheral retinal nonperfusion using widefield imaging with von Hippel-Lindau disease. Int J Retina Vitreous.

[26] Chen X, Sanfilippo CJ, Nagiel A (2018). Early Detection of Retinal Hemangioblastomas in von Hippel-Lindau Disease Using Ultra-Widefield Fluorescein Angiography. Retina.

[27] Verhaeghe I, Lafaut BA, Brandt A (1997). Retinal capillary hemangioma: von Hippel (-Lindau) disease. Bull Soc Belge Ophtalmol.

[28] Chen S, Chew EY, Chan C-C (2015). Pathology characteristics of ocular von Hippel-Lindau disease with neovascularization of the iris and cornea: a case report. J Med Case Rep.

[29] Singh AD, Nouri M, Shields CL (2002). Treatment of retinal capillary hemangioma. Ophthalmology.

[30] Rosa RH Jr, Goldberg MF, Green WR (1996). Clinicopathologic correlation of argon laser photocoagulation of retinal angiomas in a patient with von Hippel-Lindau disease followed for more than 20 years. Retina.

[31] Kolomeyer AM, Eller AW, Martel JN (2016). Spontaneous resolution of macular epiretinal membranes after fluorescein potentiated argon laser treatment of von Hippel-Lindau associated retinal hemangiomas: case report and review of literature. Retin Cases Brief Rep.

[32] Krivosic V, Kamami-Levy C, Jacob J (2017). Gaudric Laser Photocoagulation for Peripheral Retinal Capillary Hemangioblastoma in von Hippel-Lindau Disease. Ophthalmol Retina.

[33] Gaudric A, Krivosic V, Duguid G (2011). Vitreoretinal surgery for severe retinal capillary hemangiomas in von hippel-lindau disease. Ophthalmology.

[34] Chen M, Zhong L, Yao SF (2017). Verteporfin inhibits cell proliferation and induces apoptosis in human leukemia NB4 cells without light activation. Int J Med Sci.

[35] Bhattacharjee H, Deka H, Deka S (2010). Verteporfin photodynamic therapy of retinal capillary hemangioblastoma in von Hippel-Lindau disease. Indian J Ophthalmol.

[36] Kreusel KM, Bornfeld N, Lommatzsch A (1998). Ruthenium-106 brachytherapy for peripheral retinal capillary hemangioma. Ophthalmology.

[37] Raja D, Benz MS, Murray TG (2004). Salvage external beam radiotherapy of retinal capillary hemangiomas secondary to von Hippel-Lindau disease: visual and anatomic outcomes. Ophthalmology.

[38] Hussain RN, Jmor F, Damato B (2015). Verteporfin photodynamic therapy for the treatment of sporadic retinal capillary haemangioblastoma. Photodiagnosis Photodyn Ther.

[39] Garcia-Arumí J, Sararols LH, Cavero L (2000). Therapeutic options for capillary papillary angiomas. Ophthalmology.

[40] Schmidt-Erfurth UM, Kusserow C, Barbazetto IA (2002). Benefits and complications of photodynamic therapy of papillary capillary angiomas. Ophthalmology.

[41] Suzuki H, Kakurai K, Morishita S (2016). Vitrectomy for tractional retinal detachment with twin retinal capillary hemangiomas in a patient with Von Hippel-Lindau disease: a case report. Case Rep Ophthalmol.

[42] Peng CH, Cheng CK, Li SC (2010). Relaxing retinectomy for tractional retinal detachment with retinal capillary hemangiomas in genotypically confirmed von hippel-lindau disease. Retin Cases Brief Rep.

[43] Schlesinger T, Appukuttan B, Hwang T (2007). Internal En Bloc Resection and Genetic Analysis of Retinal Capillary Hemangioblastoma. Arch Ophthalmol.

[44] Avci R, Yilmaz S, Inan UU (2017). Vitreoretinal surgery for patients with severe exudative and proliferative manifestations of retinal capillary hemangioblastoma because of von Hippel-Lindau Disease. Retina.

[45] Chelala E, Dirani A, Fadlallah A (2013). Intravitreal anti-VEGF injection for the treatment of progressive juxtapapillary retinal capillary hemangioma: a case report and mini review of the literature. Clin Ophthalmol.

[46] Wong WT, Liang KJ, Hammel K (2008). Intravitreal ranibizumab therapy for retinal capillary hemangioblastoma related to von Hippel-Lindau disease. Ophthalmology.

[47] Hrisomalos FN, Maturi RK, Pata V (2010). Long-term use of intravitreal bevacizumab (Avastin) for the treatment of Von Hippel-Lindau associated retinal hemangioblastomas. Open Ophthalmol J.

[48] Karimi S, Nikkhah H, Ahmadieh H (2018). Intravitreal injection of propranolol for the treatment of retinal capillary hemangioma in a case of von Hippel-Lindau. Retin Cases Brief Rep.

[49] Knickelbein JE, Jacobs-El N, Wong WT (2017). Systemic sunitinib malate treatment for advanced juxtapapillary retinal hemangioblastomas associated with von Hippel-Lindau disease. Ophthalmol Retina.

[50] (2019). VHLA Suggested Active Surveillance Guidelines. https://www.vhl.org/wp-content/uploads/2017/07/Active-Surveillance-Guidelines.pdf.

